# The “here and now” of youth: the meanings of smoking for sexual and gender minority youth

**DOI:** 10.1186/s12954-018-0236-8

**Published:** 2018-05-31

**Authors:** Tamar M. J. Antin, Geoffrey Hunt, Emile Sanders

**Affiliations:** 1 Critical Public Health Research Group, Prevention Research Center, 180 Grand Ave, Suite 1200, Oakland, CA 94502 USA; 2 Center for Critical Public Health, Institute for Scientific Analysis, 1150 Ballena Blvd, Suite 211, Alameda, CA 94501 USA

## Abstract

**Background:**

The mainstream tobacco field in the USA tends to situate youth as passive, particularly in terms of their susceptibility to industry manipulation and peer pressure. However, failing to acknowledge youths’ agency overlooks important meanings youth ascribe to their tobacco use and how those meanings are shaped by the circumstances and structures of their everyday lives.

**Methods:**

This article is based on analysis of 58 in-depth qualitative interviews conducted with sexual and gender minority youth living in the San Francisco Bay area in California. Topics covered in interviews focused on meanings of tobacco in the lives of youth. Interviews lasted approximately 2.5 h and were transcribed verbatim and linked with ATLAS.ti, a qualitative data analysis software. Following qualitative coding, narrative segments were sorted into piles of similarity identified according to principles of pattern-level analysis to interpret to what extent meanings of smoking for young people may operate as forms of resistance, survival, and defense.

**Results:**

Analysis of our participants’ narratives highlights how smoking is connected to what Bucholtz calls the “‘here-and-now’ of young people’s experience, the social and cultural practices through which they shape their worlds” as active agents (Bucholtz, Annu Rev Anthropol31:525–52, 2003.). Specifically, narratives illustrate how smoking signifies “control” in a multitude of ways, including taking control over an oppressor, controlling the effects of exposure to traumatic or day-to-day stress, and exerting control over the physical body in terms of protecting oneself from violence or defending one’s mental health.

**Conclusions:**

These findings call into question the universal appropriateness of foundational elements that underlie tobacco control and prevention efforts directed at youth in the USA, specifically the focus on abstinence and future orientation. Implications of these findings for research, prevention, and policy are discussed, emphasizing the risk of furthering health inequities should we fail to acknowledge the “here and now” of youth.

## Background

Given evidence suggesting that most people who smoke begin during adolescence or in young adulthood [1–4], directing tobacco prevention efforts at youth is considered key for reducing long-term nicotine dependence and reducing the overall prevalence of smoking and related diseases [4, 5]. In the USA, mainstream approaches to youth tobacco use typically emphasize “risk and protective factors” and generally focus on changing individuals’ attitudes and beliefs to encourage cessation or prevent uptake of smoking [4, 6]. This may be done in several ways. For example, local community-level interventions, which work in tandem, attempt to counter personal and social factors, including stress, low self-esteem, peer pressure, and familial influences, that are putative predictors of youths’ smoking [7–9]. Such interventions include smoke-free ordinances, local anti-tobacco media campaigns, and school-, family-, and clinic-based interventions [4].

State-wide tobacco control approaches are also considered an important component of youth tobacco prevention. These include legislative and regulatory approaches that “address the social, economic, and environmental influences of tobacco use”—approaches which may extend existing local ordinances (e.g., *minimum* legal purchase age polices, smoke-free bans) or broadly implement new regulations and tobacco control efforts including increases in tobacco taxes and state-sponsored mass media campaigns designed to denormalize tobacco use and the tobacco industry among youth. Generally, these comprehensive approaches to youth tobacco prevention are considered highly effected in the movement towards a “tobacco endgame” [4].

Though these efforts are credited with significantly reducing smoking in the US general population [10, 11], smoking remains concentrated among the most disadvantaged groups [12–21], including sexual and gender minorities (SGM) [13, 22, 23]. Studies of tobacco use among SGM youth are limited [24, 25], yet available data suggest inequities similar to their adult counterparts. For example, in the 2015 Youth Risk Behavior Survey, sexual minority high school students in CA reported significantly more past-30-day cigarette smoking (~ 18%) and any past-30-day nicotine and tobacco use (i.e., cigarettes, smokeless tobacco, cigars, e-cigarettes) (~ 40%) than heterosexual students (~ 7%, ~ 27% respectively). Also, sexual minority high school students were significantly more likely to report having ever tried cigarette smoking (~ 46% compared with ~ 27%) [26]. Studies of gender minority youth are less common. However, a representative population-based study of middle and high school students in California found that transgender youth had almost five times greater odds of current cigarette smoking compared with cisgender youth, those youth whose gender identity corresponds to their sex assigned at birth [24]. Concern about the inequities inherent in who, statistically speaking, are more likely to smoke should occupy more of our focus in the tobacco field. Many scholars have highlighted the social gradient in smoking [13, 27], specifically among sexual and gender minorities [22, 23], yet tobacco control strategies remain largely focused on the general population [13, 18, 28].

A critical appraisal of two foundational components largely implicit in youth tobacco prevention strategies may be a useful starting point for investigating the causes that underlie smoking inequities among SGM youth. Take, for example, abstinence. Typically, abstinence is the only approach pursued for youth tobacco prevention in part due to concerns about the developmental risks of any tobacco use during adolescence. Though some approaches to tobacco control may be considered harm reduction strategies, most often abstinence is an explicitly-stated goal and discussions of reducing harm remain controversial, particularly when it comes to youth and young adults [29–32]. This runs counter to discourses in the prevention of drugs and alcohol where it is acknowledged that because some youth continue to use, comprehensive approaches that go beyond abstinence are necessary [33]. The controversy of harm reduction in the tobacco field arguably relates not just to the health risks presented but also concerns about the perception of “being in the service of big tobacco” [34] given the industry’s deceitful practices as well as fears that alternative approaches to tobacco prevention among youth may undermine achievements that have been made in tobacco control [29, 35]. Nevertheless, subscribing to a doctrine of abstinence, particularly given the available evidence from the drug and alcohol fields that illustrate its limitations, may only serve to reinforce smoking for some groups of youth. We cannot ignore the fact that some youth fail to “just say no” [29, 33], and that experimentation is a normal part of adolescence [29, 36–38].

A second foundational component arguably underlying mainstream youth tobacco prevention is an emphasis on future health [39, 40]. Diprose describes this type of approach as a “paradigm of preemption” shaped by a “cautious and fearful comportment toward the future it fosters” [41]. According to Keane, anti-tobacco approaches have “reduced [smoking] to its potentially most undesirable outcomes; namely, various premature, painful, and protracted forms of death” that will occur in the future [39]. The adoption of this approach for youth is surprising given extensive research documenting that “adolescents have weaker orientations toward the future, and thus they are poorer at…foreseeing long-term consequences” [42]. Therefore, for some young smokers “the seriously deleterious medical consequences of their habit lie in the future, while its rewards are experienced in the present” [39]. By focusing on future health, we are arguably failing to acknowledge youth as active agents, who also live in the ‘here-and-now’ [43], and likely, those who smoke, experience meaningful short-term benefits to smoking and place more emphasis on the perceived consequences of quitting in the present [44]. Also by emphasizing risk, smoking may become more, not less, attractive for some youth precisely because it is defined as harmful. For example, sociological research, examining the role of pleasure in drug use for youth, suggests that some youth seek to disrupt the mundane and rigid controls of everyday life by purposely engaging in “voluntary risk-taking” like drug use [45, 46]. In this same way, smoking may be one such activity where youth can “transform the routine and subvert the elements of control that take place in their everyday lives” [45].

This critique of mainstream approaches to tobacco prevention and policy warrants turning our attention to alternative approaches to studies of youths’ smoking and nicotine use—approaches that are critical in nature, highlight the agency of youth, and situate the practices of youth within a broader structural framework, considering these practices from youth’s own perspectives (see [47]). In other words, and for the purposes of this paper, what are the meanings of smoking for SGM youth themselves, particularly in the context of the structural inequities (e.g., racism, classism, sexism, homophobia) that are present in the everyday lives of some groups of young people? A significant body of social science research on youth and smoking, conducted primarily outside of the USA, highlights the unique meanings and roles of tobacco in the lives of youth, shedding light on why smoking persists despite quite widespread acknowledgement among youth about the health consequences of smoking. For example, qualitative studies have examined smoker identities among youth, illustrating the ways in which various smoker identities may be held by youth simultaneously, shifting over time and place, and that these identities are formed within a context where youth acknowledge both negative and positive meanings of smoking [6, 8, 48, 49]. Other studies have investigated the role smoking plays in identity construction more generally for youth, emphasizing youth as an authentic period of life that is not necessarily connected to adulthood [7, 40, 50–52]. Existing critical research on youth and tobacco has also centered structural inequity at its core, considering meanings of smoking with a broader structural framework, particularly emphasizing how economic disadvantage and gender, separately and at its intersections, shape meanings of tobacco and experiences with tobacco-related stigma [18, 50, 53–61]. Despite these important contributions and implications for public health, such studies appear to be largely overlooked in the tobacco field in the USA and rarely focus exclusively on the experiences of sexual and gender minority youth.

As such, more critical research on tobacco and nicotine use among SGM youth in the USA is needed if we hope to understand and prevent health inequities in smoking and related diseases. Furthermore, we would argue that more critical approaches to youth leisure practices, as developed within youth studies in general, may be useful for contextualizing and understanding the role of smoking among sexual and gender minority youth. For example, Griffin has argued that a critical perspective to youth research focuses “on the individual or collective cultural practices of particular young people as forms of resistance, defense and/or survival” [47], and by doing so, we can consider the connections between meanings of tobacco use and structural inequities experienced by SGM youth. In adopting such approaches, this paper aims to highlight the significance of situating the use of substances, in this case tobacco, within a broader structural framework. More specifically, using narrative data about smoking among 58 SGM youth in California, we will illustrate how themes of resistance, defense, and survival characterize youths’ narratives and highlight unique meanings of smoking for SGM youth. Most importantly, we will also discuss how those meanings may operate in opposition to mainstream approaches to tobacco prevention, treatment, and policy which may have the unintended consequences of sustaining inequities in smoking.

## Methods

This analysis is based on the interview narratives from youth, who participated in a larger study investigating tobacco-related stigma and meanings of smoking for 201 SGM adults living in the San Francisco Bay Area (SF Bay Area). Fifty-eight young people, between the ages of 19–25, participated in in-depth qualitative interviews which included questions on the background of the participant; social identities; initiation, practices, and pathways of smoking; beliefs about smoking; motivations for smoking; and future intentions of use. In a background closed-ended screener, 73% identified as an ethnic minority and more than a quarter of participants reported past-month housing insecurity, suggesting variation in experiences with multiple disadvantages. Research staff were highly skilled in probing techniques and posing crucial follow up questions related to main study aims which introduced flexibility to the interview process so that narratives could be generated that were participant-driven.

All study procedures were approved by the Pacific Institute for Research and Evaluation’s Institutional Review Board, and all participants were briefed on ethical procedures and provided signed documentation of informed consent prior to participating. Participants were recruited on the street, through Facebook and Craigslist advertising, and by referral. To show our appreciation for their time, participants received a $55 honorarium upon completion of the interview. Interviews lasted approximately 2.5 h and were digitally recorded. Following each interview, research interviewers completed extensive fieldnotes summarizing the interview and noting potential emergent themes and connections or conflicts with other interviews. Interview recordings were professionally transcribed and three research assistants trained in critical social research reviewed, cleaned, and then coded all transcripts to distill data into manageable analytical segments, using ATLAS.ti, a qualitative data management system [62]. During coding and to ensure an iterative approach to analysis, the research team recorded preliminary analytical ideas about the data by attaching memos to segments of interview transcripts [63]. Themes emergent from memos informed multiple codebook revisions. The final code list was comprehensive, and codes selected for this analysis included smoking behaviors, perceptions of smoking, and reasons for smoking to scope the data into manageable analytic segments. All quotations associated with these three codes were analyzed by the lead and second authors, with constant reference to the fieldnotes for each participant to ensure interpretations were made within the context of each interview in its entirety. Quotations were sorted into piles of similarity identified according to principles of pattern-level analysis, including patterns congruent or divergent with prior theory, frequency of patterns, and omission of expected patterns to interpret to what extent meanings of smoking for young people may operate as forms of resistance, defense, and survival [64, 65].

Analysis of our participants’ narratives highlights how smoking is connected to the “‘here-and-now’ of young people’s experience, the social and cultural practices through which they shape their worlds” as active agents [43]. By interpreting young people’s narratives of smoking from an analytical lens that stresses resistance, survival, and defense, we will illustrate the meanings that young people ascribe to these practices—meanings that are frequently overlooked and therefore under-emphasized in tobacco prevention, treatment, and policy. In other words, we present these three themes as conceptual frames informed by youth studies within which participant perspectives may be interpreted in ways alternative to those dominating contemporary approaches to tobacco research and policymaking. These themes are not necessarily mutually exclusive, and it will become apparent that many of the quotes from our participants could be interpreted in multiple ways. However, for the sake of argument, we discuss them separately here to help clarify our main points about the incongruence between SGM youths’ perspectives on smoking and the perspectives of the orthodoxy which inform policies designed to control tobacco use. All quotations used below are presented with pseudonyms selected by participants to humanize narratives yet maintain anonymity.

## Results and Discussion

### Resistance

The notion of resistance provides an important unifying thematic area to make sense of data that has emerged from our analysis (see [66, 67]). In examining youth cultures, researchers have considered the ways in which youth groups develop subcultures based on values opposed to or resisting the values of the dominant society. [67–69] These subcultures are considered forms of resistance through which some marginalized youth transcend negative stereotypes. [69–71] Research on youth cultures and marginalized youth has a long tradition both in the USA [72, 73] and in the UK [74–76], with much emphasis placed on the role of illicit drugs within these subcultures. [69, 77–80] While some research has examined smoking as a form of resistance for youth (e.g., [50, 67, 81]), less research has focused its examination on the ways in which tobacco use may be used by sexual and gender minority youth to cultivate an alternative definition of self-identity to resist discrimination and/or social isolation.

In one conceptualization of resistance, a clear power structure or “enemy” exists that a subgroup of youth is thought to fight against in more or less subversive ways. The “oppressor” might be patriarchy or institutionalized racism, or perhaps even a public health establishment that is perceived by some youth to be dominated by “crusaders” who do not always “tell the truth” [82]. In these cases, resistance to oppression may involve youth using “popular culture and aesthetic artifacts to fight against power” [66], and smoking may be one tool, albeit not that powerful, to exert some control over their lives through their activities. For example, SB, a 24-year-old queer woman who used to smoke, explains:

You have this radius suddenly of control, …where you’re taking up space with the smoking…, which is cool…showing passersby[s], this is the four of us. This is where we’re smoking right now. We’re talking. We’re socializing. This is kind of our area at this moment, which is really appealing for queer folks. Like, sending a message to passersby[s] who we don’t know, who might hate queers. …this is our space right now…we’re communing. We’re socializing, and we’re not alone. So, mess with us at your peril. And I’m sure there’s a big appeal to that in a lot of ways…It’s an aggression born of fear. It’s something that I say because I have been harassed as an individual for being queer…But there is a lot of strength in numbers. So yeah, […it’s] sort of a preemptive retaliation to people who would like to punish us.

Using cigarettes to control and occupy space emerged as a frequent pattern in participants’ narratives of smoking. Other scholars have highlighted meanings of smoking as control [50, 81, 83], for example, in terms of establishing a sense of control while living in circumstances of disadvantage [55, 84] or exemplifying emotional control [44, 56, 83, 85]. In our participants’ narratives, control often manifested in ways that emphasized a desire to exert control over an oppressor, like SB’s quote illustrates.

However, a clear enemy need not be articulated for a sense of resistance to be enacted [66]. For example, some participants’ narratives emphasized the deviancy socially ascribed to both queerness and smoking, powerfully linking the two so that together they functioned as a way to resist social marginalization. For example, Janet, a 25-year-old former smoker who identified her sexuality as queer, said:

For me, when I smoked, you have to go to a designated area. You’re already kind of the pariah or whatever. But, then, you bond with the other pariahs that are stuck in there. That was part of the appeal. Like, okay. Well, smokers, like, you have something to bond over by being excluded, (quick laugh) … It’s like, being gay is socially unacceptable for the longest time, but that doesn’t stop people from being gay. It just makes them form their own gay community. So, smokers are kind of, always been like their own community. Like, when I go to a group of smokers, it’s like, Yeah, yeah, yeah. I know exactly what you’re doing out here…It’s something relatable. You know?

Here, Janet expresses how smoking serves to resist social isolation and cultivate community. Literature from critical research in the alcohol and drug fields illustrates the ways in which young people use particular commodities, like substances, as cultural markers to stake out their identities in opposition to mainstream norms. The association between youth cultures and “deviant” substance use has been noted by researchers as long ago as the late 1950s when Finestone published *Cats, Kicks and Color* [86], documenting the use of heroin, dress, style, and language among young African American drug users in Chicago (for additional work on youth cultures, substances and resistance (see [87]). Critical research on tobacco has also emphasized how some youth may adopt or maintain smoking precisely because it is positioned as a deviant behavior by the same institutional structures (e.g., normative health establishment) that may already alienate youth who experience other forms of social marginalization [50, 81, 84].

### Survival

While resistance may be argued as a political response to hegemonic structures, surviving may be conceptualized as something more fundamental to life, something that is essential for getting through the day. Survival may be about an individual and their well-being, life versus death, not acting out against but instead surviving within, with no interpretation made about behaviors as related to resistance. Of course, some scholars have argued that just by virtue of surviving one “can signify a form of resistance” in an oppressive culture [47]. Nevertheless, and for the sake of argument, narratives from our studies illustrated several ways in which survival and smoking may be connected. The first is emotional survival.

Though youth often discussed potential long-term consequences of smoking, the short-term benefits associated with smoking for getting through the day-to-day, in terms of daily stress and anxiety, often outweighed concerns about future health. For example, Gigi, a 25-year-old trans woman who is trying to cut back on her smoking, noted:

Stress that I feel like I can’t control because it’s dependent upon another person, or another situation that is larger than what I have in my control…Because while I know that I can’t control it, I still desire to have answers, or to be able to control it. And I know that’s one thing that makes me want the enjoyment that comes from smoking. I know, [smoking] at least temporarily relieves that feeling, that’s what I associate it with.

Other participants’ narratives situated their smoking in terms of emotional survival within the context of coping with traumatic stress associated with everyday experiences with discrimination and marginalization. For example, Jen a 22-year-old former smoker, who identifies her sexuality as bisexual, talks about the value of smoking to survive within a heterosexist society. She plainly situates smoking as a tool for survival, something supported in literature on smoking among women living in circumstances of disadvantage [55].

[LGBTQ] people’s lives are really hard. Just going through it and making the most of it would probably mean having a cigarette once in a while, because I’m just going to do what I want in life. If what I have gone through hasn’t killed me so far, …the cigarette is probably not going to kill me. So it’s not really a high priority for a lot of people to think about…I think if you are not out with your family…If you have to act straight to get through…your friends, your family or colleagues, I think that adds a lot of stress to your life. And yeah, just seeing the kind of violence that is out there against LGBTQ people. It’s a really sad and emotional thing…So I think they are much more sensitive to that. Probably a lot more inclined to just want to push that to the back of your mind and smoke a cigarette, get rid of the ideas and move on with their lives.

Similarly, a 23-year-old current smoker, who self-identified as a gay cisgender man but did not provide a pseudonym, described a salient discriminatory experience in a clothing store around the age of 17 when he was trying on dresses for a school dance.

I picked up one of the long dresses. I said, ‘*ma’am, can I try this on in the changing room? I want to see if this is gonna be my size, if it fits me*’ … She said, ‘*excuse me?*’ ‘*Well yeah, I want to try this on. I’m getting ready for prom. We’re here picking out dresses*’. She said, ‘*no, I can’t let you do that. These are for women’s only.*’ Oh okay. I could have put that on the news, real quick. There would have been a whole problem, and she probably could have lost her job for discriminating against me….But, I decided to just put on the dress anyway. I was like ‘*oh, it don’t fit (laughs). I think it’s gonna rip. Can you help me?*’ The woman didn’t want to help. My friends were there, just laughing. We’re all laughing….It’s crazy to see how people are so close minded or judgmental, or non-accepting of someone who wants to express themselves as who they are…My feelings weren’t hurt, but I’m pretty sure someone else in that store, feelings could have been hurt, or someone could have been offended. And that’s the sad part. That made me want a cigarette. Like damn, you are that fucked up, to feel that way towards me. And to be rude towards me. You stressed me out. Now I need a cigarette.

Day-to-day stressors, varying in degree of severity in terms of their perceived consequences for mental and physical health, saturated participants’ narratives and were often explicitly linked to a need for smoking to cope.

Intimately connected to emotional survival is pleasure, which is a rarely discussed attribute of smoking despite its tremendous importance for smokers (e.g., [88]). For example, SB, introduced above, explained:

Being queer in a heterosexist society is very stressful. I’m willing to bet -- in fact, I can tell you definitively that a lot of substance abuse within the queer community is directly tied to that stress, to that sense of comfort and support that is difficult to find outside [in] the big brawn scary world. […] Just a sense of: this is something I can control. It feels good. I can come back to it. I have control over it. It’s something I can kind of take with me, when I go out into public. I can still carry the feeling at least…It’s addressing I think stresses and anxieties and self-loathing that we’re socialized to accept in ourselves […] I can’t change the society around me, but I can change the way I feel. So, it was a misguided attempt to really take control over how I felt in that society that seemed unwelcome of me.

Here, SB smokes because it “feels good” and she can “carry” that pleasurable experience as a possible protection from hate and as a palliative for “stress” and “self-loathing”. In the 1980s and 1990s, scholars began to investigate pleasure and youth subcultures, where pleasure becomes a way to avoid or overcome the mundane of everyday life [89–92]. However, here and in the narratives of other participants, smoking as pleasure goes beyond just overcoming the mundane nature or routinization of everyday life, but also was emphasized as a tool for experiencing some pleasure within an inequitable and oppressive society that feels beyond one’s control.

Participants’ narratives also illustrated survival in terms of surviving socially. Literature in the tobacco field often talks about youths’ smoking in more passive terms, specifically by emphasizing smoking as a result of peer pressure. The implications of this interpretation, then, often results in individual-level prevention efforts that “focus on cognitive factors that mitigate the effects of peer group influences” [93]. However, narratives from our participants were more active, illustrating how smoking was less connected to ‘I smoke because my friends smoke’—though that was present in some narratives—and more connected to ‘I smoke to survive in social situations’. For example, in the next quote, we further see how Jen, quoted above, also strategically used smoking to connect with others.

My school was super conservative, really Christian…It was the exact opposite of me. So when I moved there, I was really just reaching out to anybody who had any progressive, liberal in their body and anyone who is atheist. What is kind of interesting, the ones that were more my type of people to talk to and have conversations with, smoked. So that was something I ended up picking up just to talk with them….

The emphasis here is less on peer pressure but instead on group solidarity and group identification. Of course, there is a literature supportive of this notion of sharing commodities and “intoxication” with others (see [94–96]) where the focus is not on peer pressure but instead on the sociability that is shared when a substance is consumed together. These are two very different interpretations on the role of substances of course, and these different interpretations are important, because while one emphasizes the agency of youth, the other sees young people as passive and easily able to succumb to peer pressure (for a further discussion and critique of notions of peer pressure see [97]).

In a context of social marginalization, the importance of group belonging also takes on additional meanings for our participants, where smoking facilitates entrée into certain groups where social acceptance is more likely. Similarly, in their study of disadvantaged and socially marginalized youth in Australia, Hefler and Carter [58] found that smoking served as a way for some socially stigmatized youth to adopt a “compromise” identity in what they perceived to be a less than ideal social context but which for some youth nevertheless “provided some sense of belonging” (p 11).

A mainstream and rationalistic interpretation of these narratives of survival might only consider smoking as a poor decision for coping with stress during this universal life stage that tends to be essentialized as a period of “stress and storm” [43, 98]. However, we would argue that it is also important to remember that youths’ experiences exist in the “here-and-now” and that for some youth, smoking is a particularly useful tool for alleviating feelings of anxiety and stress, particularly those stemming from discriminatory treatment and trauma. As such, emphasizing future health in tobacco control and prevention may do nothing to counter the value that some youth place on smoking for surviving and getting-by in the present.

### Defense

Finally, narratives of defense appeared frequently in discussions related to youth perceptions about and reasons for smoking. In critical youth studies, discourses of defense (and survival, for that matter) emerged in response to critiques of resistance theory which argued that researchers’ interpretations of social practices as forms of resistance “imbued [them] with magisterial authority” and “carried the possibility of romanticizing specific cultural practices as ‘resistant’ which might also be sexist or racist or both” [47, 99]. When avoiding speculation about whether some of our participants’ narratives about smoking illustrate acts of resistance, patterns of defense emerged illustrating how smoking is used strategically as a form of self-protection. For example, participants often discussed smoking as a way of creating “safe” space around them to protect themselves from physical violence and harassment. For example, Marisol, a 22-year-old queer woman said:

…if I go out and I’m dressed up really femme and people will usually think like, she can’t defend herself or whatever. And I feel like when I smoke cigarettes – obviously there is this idea that you look tougher, that you can actually beat someone up, even though that is not true. So, I think – if someone is harassing me or if I want to scare someone away, for some reason I feel like smoking a cigarette will be like, ‘don’t mess with me.’ You know?... I could do this when I’m at a straight bar and I’m surrounded by straight people and straight men are harassing me and I’m just trying to basically make it seem like I could handle myself, so get away from me.

Participants frequently described how they capitalized on the symbolic meanings associated with smoking—e.g., as is the case with Marisol above, smoking as a sign of strength and toughness [44, 56, 83]—to protect their gendered bodies by creating symbolic “safe” spaces where they could more easily defend themselves from potential harassment. Though some research has emphasized the creation of spaces accepting of smoking in response to the stigmatization of smoking and arguably the smoker [8, 54, 60, 100], few studies have illustrated how smokers strategically use smoking to transform, at least partially, “unsafe” spaces into “safe” spaces as defense against homophobia or sexism. Relying on smoking for protection emerged not only for sexual minority women in heteronormative spaces, but also for women in spaces defined by gay men and for gender non-conforming participants in a multitude of contexts due to everyday threats of violence. A material object can shift “how an individual relates to and moves through “unsafe” space” [101]. In their synthesis of the literature on “safe spaces”, the Roestone Collective [101] argues that objects (like cigarettes for our purposes) can “alter the constitution of and possibilities for safe spaces,” and offer at least “incomplete solutions” for defending against the oppressive structural conditions in which some people find themselves (p 1360).

Narratives of defense also emerged with respect to participants’ desires to protect their own health through the very act of smoking, a perspective that at first glance conflicts with normative conceptualizations of health and how best to protect it. For example, Ana, a 20-year-old current smoker who identifies their^1^ gender as nonbinary trans and their sexuality as queer, explained:

Working class people, folks of color and queers and god forbid if you are all three of those things, you are going to be smoking. You are stressed out. There are not a lot of things that are accessible for you in terms of relief. Like, who can afford to get a massage every week? I can’t. Who can afford to get mental health care? Sometimes smoking a cigarette is the difference between…cutting myself or not. If I give myself that ten-minute break, I don’t do that reactionary thing. So sometimes I think it is a coping mechanism. Sometimes it’s the only one and it’s the best one that people have.

While participants in general were not unaware of the health risks that smoking posed, they nevertheless stressed the importance of smoking for mitigating serious mental health risks that they faced in the present. Sociologists involved in research on youth and substance use (tobacco, alcohol, and illicit drugs) have emphasized the tendency by researchers to portray young people as passive and risky and, therefore, irrational. Consequently, youth are often considered in need of protection from becoming the “victims of their own irresponsibility” [102]. However, given the variety of risks that some young SGM participants may find themselves facing in the “here and now”—such as mental health crises, sexist violence, or lack of access to health resources—smoking for these young people could instead be understood as an active and quite rational response. Thus, participants’ narratives from this study highlight they ways in which these youth prioritize meaningful short-term benefits associated with smoking, in defense of their physical and mental health, over the long-term physical health consequences that smoking may pose. Indeed, for many participants, negotiations around smoking and health consequences involve considerations of well-being that are much more complex and relative than can be recognized from the perspectives currently dominating tobacco control approaches.

## Conclusion

Analysis of our participants’ narratives highlights how smoking is connected to the “‘here-and-now’ of young people’s experience, the social and cultural practices through which they shape their worlds” as agents [43]. As Hughes argues in his analysis of the “long-term development of tobacco use in the West,” contemporary tobacco use is largely considered “as an instrument of self control” [83]. Our studies of disadvantaged youth further justify this role of tobacco, where smoking signified control in a multitude of ways, including taking control over an oppressor, controlling the effects of exposure to traumatic or day-to-day stress, and exerting control over the physical body in terms of protecting oneself from violence or defending one’s mental health. Tobacco prevention, treatment, and policy seldom acknowledges these meanings and the perceived benefits that youth associate with their tobacco use, instead situating youth as passive actors. Such an oversight, however, risks overlooking how tobacco use is grounded in youths’ everyday lives and not necessarily in their concern for their futures. We have presented participants’ narratives about their own smoking not from a perspective that situates tobacco use as a social and health problem but, instead, from a perspective that seeks to understand these practices from youth’s own perspectives and in concert with the structural context in which these youth live. Sexual and gender minority youth may ascribe radically different meanings to smoking compared with youth who experience more advantages in their day-to-day lives. However, if we hope to reduce inequities in smoking, these meanings must also be taken into consideration.

By taking a critical approach to our studies of youth smoking, it becomes clear how foundational elements underlying approaches to youth tobacco prevention and cessation, i.e., abstinence and an orientation to the future, specifically future health, may not necessarily resonate with all young people, particularly those who smoke because of the important perceived benefits that they can experience now. In fact, it may be the case that as long as tobacco prevention efforts continue to position smoking as a socially unacceptable practice and a threat to future health, some youth will remain drawn to smoking, either because messages fail to resonate with them or because the risks of stopping smoking at the moment feel greater than the risks smoking poses for future health [44, 81].

Not only is more research needed that takes a critical approach to studies of youth and tobacco in the United States, but it is also important that this more critically orientated research is a part of the conversation in developing innovative tobacco prevention and policy efforts that are sensitive to the experiences of youth who continue to smoke, including SGM youth. Otherwise, we risk furthering the existing inequities in smoking. Perhaps this means making structural inequities and oppression a tobacco control issue. Perhaps this means explicitly pursuing harm reduction—where not all tobacco and nicotine products are treated as equally harmful—over abstinence in youth tobacco prevention, rather than remaining so focused on a “tobacco endgame” that we ignore the role that smoking plays in young people’s lives [32, 50, 103, 104]. Qualitative research suggests that vaping, for example, may be perceived by smokers to be an effective transitional tool for moving towards smoking cessation [105, 106], even for youth [29, 107, 108]. Yet to date, too little research has investigated to what extent vaping could serve as a suitable and potential replacement for smoking for youth, an omission perhaps explained in part by the negligible role of critically oriented tobacco research in policy and practice. However, if we hope to reduce inequities in smoking, “it is past time to add new and even radical approaches” ([29], p 14) and work towards a reality where the few who persist in smoking at least do so on an equitable playing field.

## Footnotes

1 Ana’s preferred pronouns are they/them/their.
